# Low temperature modulates spike development and yield *via* ABA pathway at post-reproductive transition stage in wheat

**DOI:** 10.1016/j.abiote.2026.100086

**Published:** 2026-08-25

**Authors:** Ankui Liu, Siqi Wu, Sulaiman Saeed, Mengxin Wang, Xindi Wang, Haotian Tu, Wenhao Yan, Chao He, Yequn Wu

**Affiliations:** National Key Laboratory of Crop Genetic Improvement, College of Bio-X, Hubei Hongshan Laboratory, Huazhong Agricultural University, Wuhan, 430070, China

**Keywords:** Wheat (*Triticum aestivum* L.), Low temperature, Reproductive transition, Spike development, Abscisic acid

## Abstract

Wheat (*Triticum aestivum* L.) yield is very sensitive to environmental temperature. Here, we integrated field sowing experiments and controlled-temperature treatments to explore how low temperature affects spike differentiation and yield formation in wheat. Early sowing allowed the wheat to undergo the reproductive transition before the winter, supporting prolonged spikelet differentiation under low-temperature conditions. By contrast, late sowing markedly shortened the reproductive growth period, reducing effective tiller number per plant and grain number per spike. Exposure to low temperature after the reproductive transition (Vs) significantly delayed heading while increasing spike length, the number of fertile spikelets, and the number of grains per spike. Temperature-gradient experiments (8, 16, and 22 °C) revealed that abscisic acid (ABA) signaling functions as a central hub integrating temperature cues: ABA signaling activates plant defense against cold at 8 °C, maintains developmental homeostasis at 16 °C, and is suppressed at 22 °C to prioritize reproductive growth. Integrated transcriptomic and plant hormonal analyses during floret differentiation showed that Vs treatment triggered extensive transcriptional reprogramming, with significant enrichment of phytohormone signaling pathways. These observations reveal that exposure to low temperatures after the reproductive transition, rather than vegetative vernalization, optimizes yield potential through ABA-mediated transcriptional reprogramming and crosstalk among multiple plant hormones. This provides a theoretical basis and molecular targets for optimizing sowing time and breeding climate-resilient wheat varieties under global climate change.

## 1 Introduction

Fluctuations in temperature patterns, such as warm winters and late spring cold spells, are increasingly disrupting the phenological development of wheat (*Triticum aestivum* L.), posing a serious threat to final yields [1]. Spike development largely determines wheat yield and this process is extremely sensitive to environmental temperatures [2,3] including low temperatures.

The effects of low temperatures on wheat are stage dependent [4]. During the seedling and vegetative stages, vernalization, i.e., long exposure to cold, promotes the transition to flowering in winter cereals [5]. During the reproductive phase, however, low temperatures have adverse effects on spike development and grain yield, decreasing the grain number per spike [6] and causing floret degeneration [7].

Most studies on the role of temperature in regulating wheat development have focused on vernalization during vegetative growth [8]. As a classic low-temperature adaptation, vernalization regulates the expression of flowering-related genes to ensure that plants enter reproductive growth only after experiencing winter cold. Prior to cold exposure, Vernalization 2 (VRN2) represses *VRN3* expression to maintain vegetative growth; upon vernalization, VRN1 accumulates and represses *VRN2* expression, thereby derepressing *VRN3* and initiating a positive feedback loop that locks the plant into reproductive development [5]. Recent epigenomic studies have revealed that the vernalization-induced activation of *VRN1* involves chromatin remodeling characterized by reduced H3K27me3 and increased H3K4me3 at the *VRN1* promoter and that Squamosa promoter-binding protein-like (SPL) transcription factors (TFs) regulate this process [9]. In field experiments, there were significant differences in yield performance between early sowing and late sowing even for semi-winter varieties that had fulfilled the vernalization requirement [[10], [11], [12]], suggesting that the temperature environment after the reproductive transition may independently regulate panicle development.

The transduction of temperature signals relies on the integration of the plant hormone network. Abscisic acid (ABA) serves as a central mediator of cold stress responses, and its biosynthetic and signaling components are dynamically regulated during exposure to low-temperature [13,14]. In addition, jasmonic acid (JA), salicylic acid (SA), and auxin (indole-3-acetic acid, IAA) have been implicated in whole-plant or leaf-level cold tolerance [[15], [16], [17], [18], [19]]. To date, most studies of phytohormones have focused on whole plants or leaves. The spatiotemporal effects of low-temperature and phytohormone signaling during the development of the young spike, which is directly involved in yield formation, remain unclear. Furthermore, wheat varieties with different vernalization requirements significantly differ in their responses to temperature changes [20]. How genotypic differences affect perception of low temperatures and yield-related trait formation in wheat also remains to be explored.

In this study, we investigated how low temperature during spike differentiation modulates yield formation in wheat, using sowing-date field experiments and controlled-temperature treatments to independently assess temperature effects. We determined that a low-temperature signal following the reproductive transition delays the differentiation of young spikelets by activating an ABA-centered plant hormone transduction network, thereby promoting spike development and increasing the grain number per spike. Winter- and spring-type wheat varieties exhibited different responses to temperature due to variations in sensitivity to phytohormone signals. We conclude that genetically enhancing the low-temperature responsiveness of wheat at the reproductive stage, rather than merely adjusting the sowing date, offers a robust strategy for obtaining stable high yields under fluctuating climate conditions.

## 2 Results

### 2.1 Sowing date affects the growth and yield-related traits of wheat

**Fig. 1:**
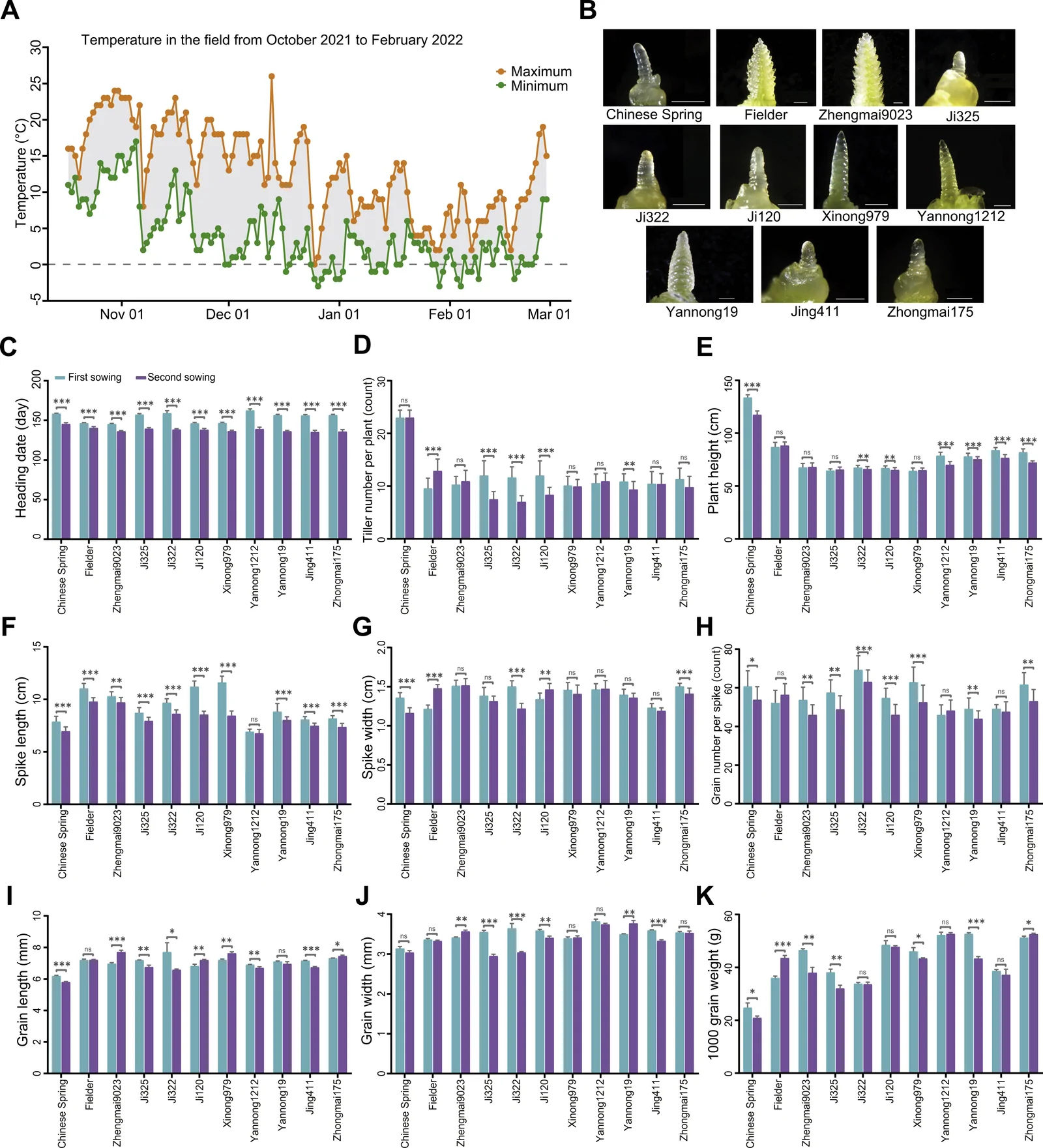
Early sowing promotes spike development and enhances wheat yield potential under low-temperature conditions. **A** Daily temperatures from sowing to vernalization. **B** Microscopic observation of young panicles from early-sown plants under a stable daily maximum temperature of 10 °C, bar = 0.5 mm. **C-K** Effects of sowing date on heading date (**C**), tiller number per plant (**D**), plant height (**E**), spike length (**F**), spike width (**G**), grain number per spike (**H**), grain length (**I**), grain width (**J**), and thousand grain weight (**K**) of different wheat varieties. The data represent the mean ± SD; significant differences were determined by a two-tailed Student's *t*-test, ∗*P* < 0.05, ∗∗*P* < 0.01, ∗∗∗*P* < 0.001, ns: not significant. Each data point was derived from three sets of biological replicates, each containing ten samples.

Sowing date strongly influences the timing of the reproductive transition and subsequent spike development in wheat. To investigate the effects of low temperatures at different stages of development on wheat yield, we selected 11 wheat varieties with varying winter and spring characteristics as materials (Table S1). We conducted field sowing in batches on October 17 and November 4, 2021 and observed young spike development when the daily maximum temperature remained at ∼10 °C (Fig. 1A and Table S2).

Sowing date significantly altered the relationship between spike differentiation and environmental temperature: early-sown materials (mid-October) entered the reproductive transition stage before the peak of low winter temperatures, allowing spikelets to complete differentiation under prolonged winter cold conditions (Fig. 1B). By contrast, late-sown materials (early November) continued vegetative growth throughout the winter, with spikelet differentiation primarily occurring after temperature recovery in the spring (Fig. S1A).

Late sowing significantly shortened the reproductive growth period of wheat (Table S3). Despite an over two-week difference in sowing date, the difference in heading date for most varieties was less than 5 days (Fig. 1C). This shortened developmental duration had a significant negative impact on yield-determining traits: except for the typical spring-type variety Fielder, late sowing resulted in decreased effective tiller number per plant (Fig. 1D) and plant height (Fig. 1E) across most varieties (e.g., a 30.4%–39.9% reduction in the Ji Mai series).

Spike traits and yield components exhibited different responses to sowing date. Late sowing generally resulted in a significant reduction in spike length in most varieties, along with a decrease in the number of fertile spikelets, the number of grains per spike, and the thousand grain weight (TGW). However, in Fielder and Zhongmai 175, the TGW increased under late sowing (Fig. 1F and K, Fig. S1). For common varieties in the wheat-growing areas of Hubei, early-sown Zhengmai 9023 (weak spring type) and Xinong 979 (semi-winter type) outperformed late-sown plants in terms of both key spike traits and final yield (Fig. 1F and K, Fig. S1). Therefore, even spring-type varieties exhibited higher yield potential when exposed to low temperatures after the reproductive transition.

Notably, the magnitude of these responses to different sowing dates was closely associated with the winter/spring growth habits of the 11 varieties (Table S1). Semi-winter varieties exhibited particularly pronounced responses to delayed sowing, with decreased tiller number per plant, spike length, fertile spikelet number, and grain number per spike. By contrast, spring-type varieties displayed relatively moderate changes across these traits, with Fielder even showing an increase in TGW under late-sowing conditions (Fig. 1K). These differential responses are consistent with the previous finding that winter and semi-winter wheat require a prolonged low-temperature vernalization period to complete floral induction [21,22]. Thus, early sowing extends the duration of young spike differentiation, facilitating assimilate accumulation and sufficient spike development, whereas late sowing shortens the reproductive growth phase, ultimately reducing yield.

Our observations indicate that sowing date significantly affects the growth and yield-related traits of wheat by altering its initial growth environmental temperature. Furthermore, winter and spring wheat varieties exhibited different responses to environmental changes. For winter and semi-winter varieties, experiencing sufficient low temperature during the young spike differentiation stage is critical for robust spike development and yield potential. By contrast, spring-type varieties exhibit greater flexibility, with some maintaining or even improving certain yield components under shortened cold exposure during this stage.

### 2.2 Young spike development and yield-related traits in wheat of different vernalization types depend on temperature

**Fig. 2:**
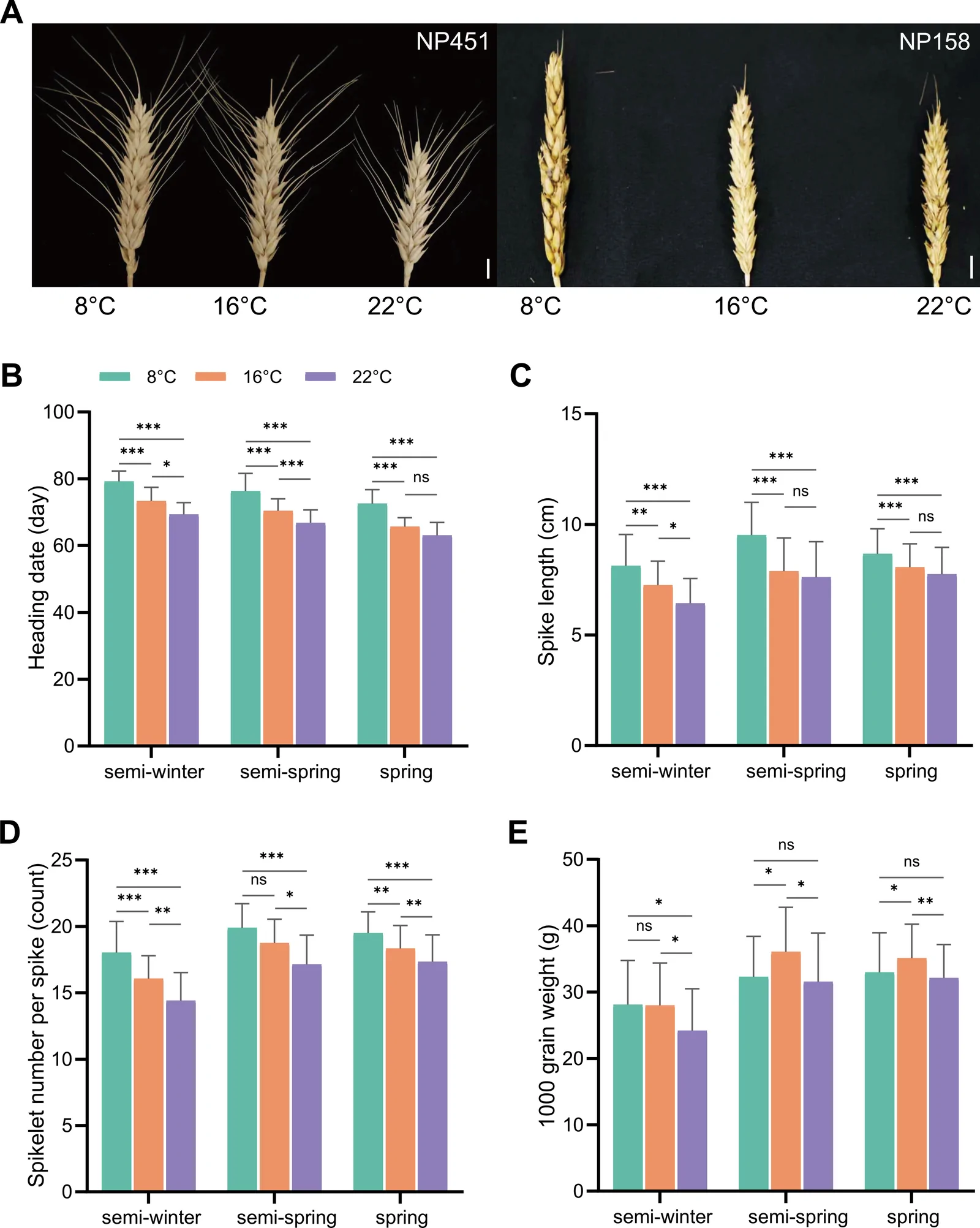
Temperature responses of spike development and yield traits depend on wheat vernalization requirements. **A** Spike phenotypes of representative wheat varieties under different temperature treatments, bar = 1 cm. **B** Heading dates of semi-winter, semi-spring, and spring varieties under different temperature treatments. **C** Spike length of semi-winter, semi-spring, and spring varieties under different temperature treatments. **D** Spikelet number per spike of semi-winter, semi-spring, and spring varieties under different temperature treatments. **E** Thousand grain weight of semi-winter, semi-spring, and spring varieties under different temperature treatments. The data represent the mean ± SD; significant differences were determined by one-way ANOVA, ∗*P* < 0.05, ∗∗*P* < 0.01, ∗∗∗*P* < 0.001, ns: not significant.

Wheat varieties with contrasting vernalization requirements exhibited distinct developmental and yield responses to defined temperature regimes. To examine the precise mechanism by which temperature regulates spike development and yield, we examined the phenotypes of 121 diverse wheat varieties grown under a temperature gradient in a greenhouse. After two weeks of exposure to 8, 16, or 22 °C during the double-ridge stage, semi-winter, semi-spring, and spring varieties exhibited significantly different developmental responses (Fig. 2A and Table S4).

Semi-winter wheat exhibited significantly delayed heading at 8 °C but produced the highest grain number per spike, indicating its reliance on low temperatures to complete vernalization and optimize grain setting in spikes (Fig. 2 and Fig. S2). At 22 °C, spring wheat had the earliest heading and the largest grain number per spike, but at 8 °C, its development was slightly inhibited, indicating that it basically did not require vernalization. Semi-spring wheat showed intermediate performance, with an optimal TGW at 16 °C. The spike length decreased with increasing temperature, with the longest spikes at 8 °C and the shortest at 22 °C. However, semi-winter wheat exhibited shorter spikes but denser grains at low temperatures, indicating that low temperature promotes floret differentiation rather than spike elongation. In summary, wheat with different vernalization requirements showed significantly different responses to temperature at the double-ridge stage. Temperature not only regulates heading time, but also profoundly affects yield.

To systematically analyze how temperature regulates young wheat spike development, we performed transcriptome sequencing of wheat spikelets from temperature-sensitive and temperature-insensitive wheat varieties after treatment at 8, 16, and 22 °C (Fig. S3A and B, Table S5). The changes in gene expression of temperature-sensitive and temperature-insensitive wheat varieties varied at specific temperatures (Table S6 and Table S7). Notably, genes specifically induced at 16 °C in the temperature-sensitive group (C2) were significantly enriched in the gene ontology (GO) term ABA-activated signaling pathway (GO:0009738, Fig. 3A and S3C). Therefore, we investigated the expression patterns and regulatory relationships of ABA-associated genes under different temperature conditions. Motif-supported regulatory network analysis uncovered the hierarchical architecture underlying temperature-responsive transcription. By ranking TFs according to outdegree centrality, we identified the top 10 hub non-ABA-related regulators in each co-expression cluster of both temperature-sensitive and temperature-insensitive genotypes, including *ERF*, *NAC*, *WRKY*, *MYB*, *TCP*, and *bZIP*, which exhibited markedly divergent regulatory effects across clusters and between genotypes (Fig. S3D).

**Fig. 3:**
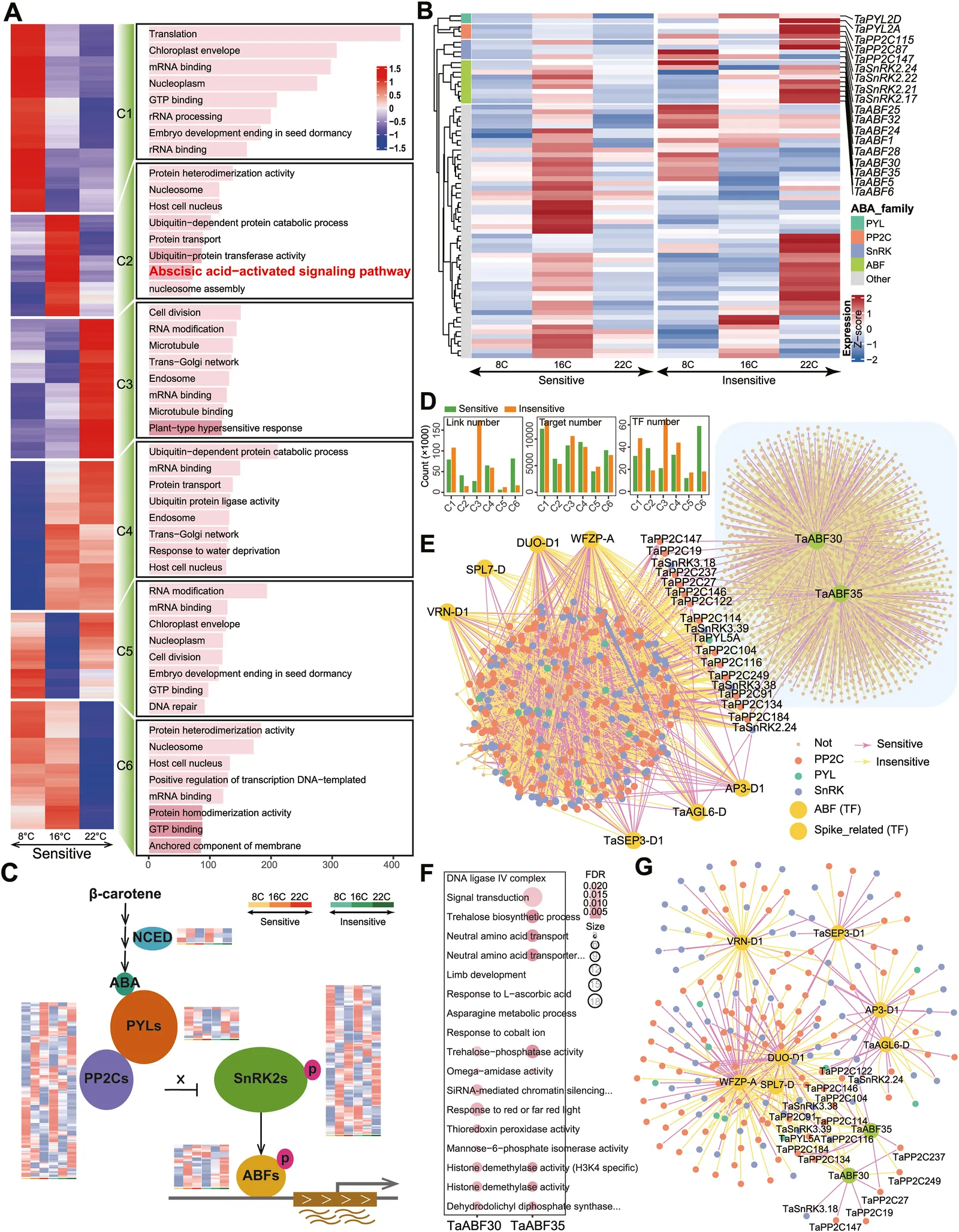
ABA signaling acts as a central regulatory hub in temperature-responsive spike development. **A** Clustering of temperature-responsive genes in the sensitive wheat variety across 8, 16, and 22 °C treatments, based on expression patterns. Genes with TPM < 0.5 at any temperature were excluded prior to clustering. Six expression clusters (C1–C6) were identified; representative enriched Gene Ontology (GO) terms for each cluster are shown on the right. The abscisic acid-activated signaling pathway (GO:0009738) was significantly enriched in cluster C2. **B** Heatmap showing the expression profiles of genes enriched in the abscisic acid-activated signaling pathway in sensitive wheat varieties under three temperature treatments. Previously reported ABA-related gene families, including *PYL*, *PP2C*, *SnRK*, and *ABF*, are indicated. **C** Heatmaps of the expression patterns of representative ABA pathway gene families, including *NCED*, *PYL*, *PP2C*, *SnRK2*, and *ABF*, in temperature-sensitive and temperature-insensitive wheat varieties across the three temperature conditions. **D** Summary of the motif-supported candidate regulatory networks constructed for each expression cluster in sensitive and insensitive wheat varieties, showing the numbers of regulatory links, target genes and the corresponding upregulated network. **E** Global motif-supported candidate gene regulatory network for genes involved in ABA signaling. Nodes represent TFs or target genes, and edges represent predicted TF–target regulatory relationships supported by promoter motif enrichment and consistent expression patterns. Hub TFs are highlighted. **F** Gene ontology enrichment analysis of the predicted downstream target genes of the hub TFs TaABF30 and TaABF35. **G** ABA-related regulatory subnetwork extracted from the global candidate regulatory network, showing the predicted regulatory relationships among ABA pathway genes and their upstream TFs.

To further characterize the enriched ABA-responsive genes, we extracted all genes assigned to this pathway and compared their expression patterns between the two wheat ecotypes (Fig. 3B, Table S8) [[23], [24], [25], [26], [27]]. Besides genes of unknown function, this gene set contained genes encoding multiple well-characterized ABA signaling components, including members of the *PYL*, *PP2C*, *SnRK*, and *ABF* gene families. Most of these genes displayed a pronounced 16 °C-specific induction in the temperature-sensitive variety, whereas many genes were more highly expressed at 22 °C in the temperature-insensitive variety, indicating distinct transcriptional responses of ABA signaling components to temperature in the two wheat ecotypes. We then examined the expression patterns of representative genes from the major ABA signaling modules, including *NCED*, *PYL*, *PP2C*, *SnRK2*, and *ABF* (Fig. 3C). Although genes within the same family exhibited diverse expression patterns, substantial differences were observed between the two wheat ecotypes, suggesting that multiple components of the ABA pathway are coordinately reprogrammed during temperature-dependent young spike development.

To investigate the upstream regulatory mechanisms underlying these temperature-responsive transcriptional changes, we scanned the promoter regions of genes from each expression cluster for enriched TF binding motifs using FIMO. Candidate TF–target relationships supported by both the occurrence of motifs in gene promoters and consistent expression patterns were retained to construct motif-supported regulatory networks (Fig. 3D and E). The resulting networks comprised 299,640 and 384,673 candidate regulatory interactions in the temperature-sensitive and temperature-insensitive varieties, respectively. Despite involving only dozens of TFs, each network regulates thousands of downstream genes, indicating highly hierarchical transcriptional regulation. Among these, the ABA-related TFs TaABF30 and TaABF35 were identified as major hubs with extensive downstream targets. GO enrichment analysis of the predicted downstream targets of TaABF30 and TaABF35 pointed to the distinct biological functions of these two hub TFs (Fig. 3F). The predicted targets of TaABF30 were significantly enriched in responses to environmental stimuli, carbohydrate metabolism, and epigenetic regulation. The predicted targets of TaABF35 were preferentially enriched in signal transduction, neutral amino acid transport, trehalose biosynthetic process, and histone demethylase activity. Furthermore, extraction of the ABA-associated subnetwork demonstrated that ABA biosynthesis, perception, signal transduction, and transcriptional regulation are extensively connected with other developmental regulators involved in young spike development (Fig. 3G) [28], supporting a central role for ABA-associated regulatory modules in coordinating temperature-responsive transcriptional programs.

To validate the temperature-responsive expression profiles, we examined the expression of five candidate genes (*MKKK70-A*, *bZIP62-D*, *HAP2G-B*, *PYL5*, and *SAPK10*) in sensitive and insensitive wheat materials at 8, 16, and 22 °C using RT-qPCR. The expression patterns of all genes examined by RT-qPCR were consistent with the RNA-seq data in both genotypes across the three treatments (Fig. S4), further supporting the reliability of the transcriptomic data. The differences in temperature response strategies of ABA pathway genes between the two genotypes suggest that the ABA signaling pathway plays a central regulatory role at all temperatures.

### 2.3 Low temperature positively regulates spike development and yield after the reproductive transition

**Fig. 4:**
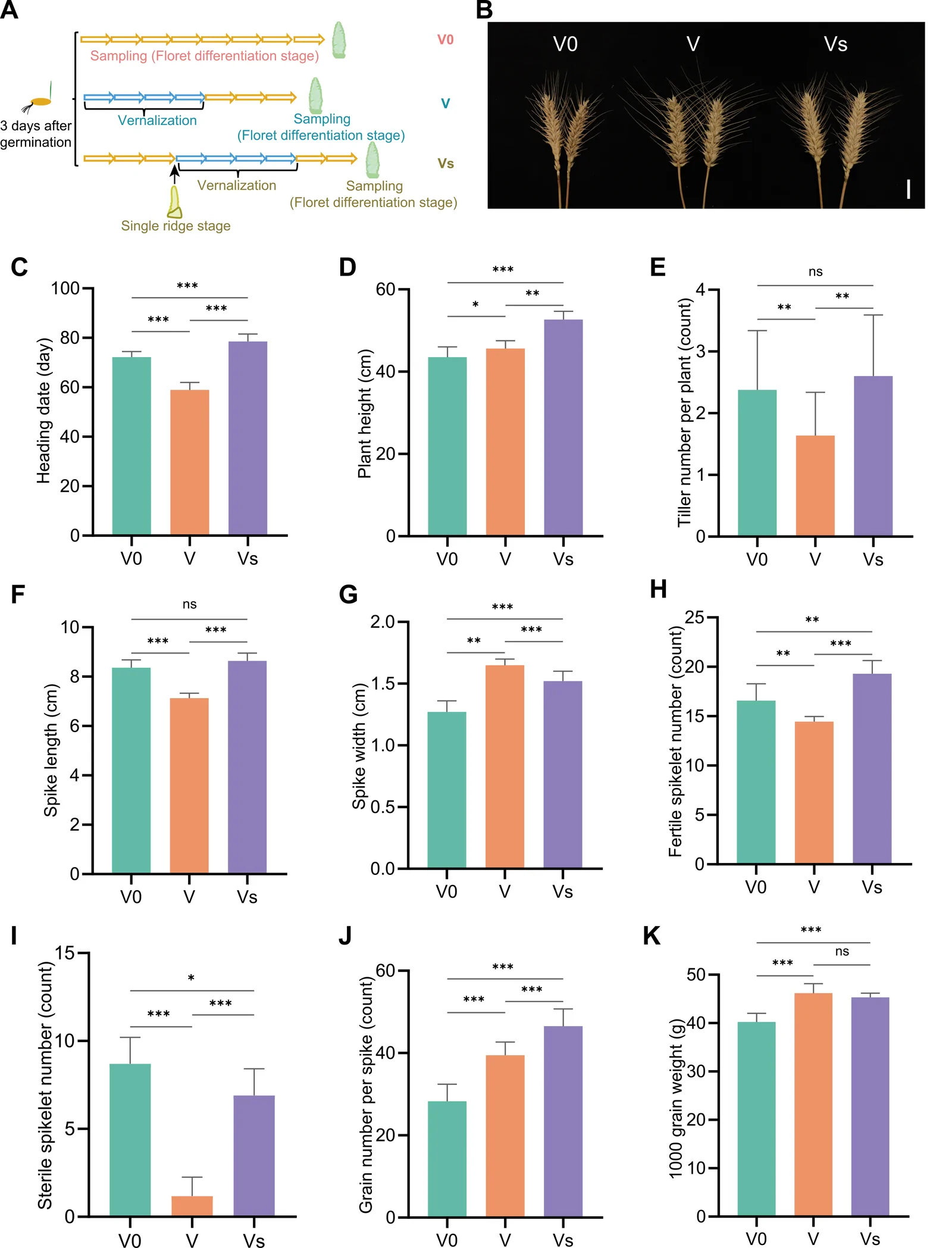
Low-temperature exposure after the reproductive transition promotes spike development and increases yield-related traits. **A** Diagram of low-temperature treatment of wheat at different stages of growth. **B** Spike phenotypes of Ji 325 under low-temperature treatment at different stages of growth, bar = 1 cm. **C-K** Heading date (**C**), plant height (**D**), tiller number per plant (**E**), spike length (**F**), spike width (**G**), fertile spikelet number (**H**), sterile spikelet number (**I**), grain number per spike (**J**), and thousand grain weight (**K**) of Ji 325 under low-temperature treatment at different stages of growth. V0: Without vernalization. V: Vernalization during the vegetative stage. Vs: Low-temperature treatment after the initiation of the reproductive transition. The data represent the mean ± SD; significant differences were determined by one-way ANOVA, ∗*P* < 0.05, ∗∗*P* < 0.01, ∗∗∗*P* < 0.001, ns: not significant.

Based on the significant sensitivity of wheat yield to temperature variations, we established three treatment groups to investigate the impact of spatiotemporal differences in low-temperature responses on spike morphology and yield in wheat (Fig. 4A): no low-temperature treatment (V0), low-temperature treatment during the vegetative stage (V), and low-temperature treatment after the reproductive transition (Vs). Given the sensitivity of the semi-winter variety Ji 325 to sowing date observed in the field experiments, we selected Ji 325 as the experimental material.

After the reproductive transition, young spikelets were highly sensitive to low-temperature signals (Fig. 4B). Compared with the V0 group, V treatment significantly accelerated development, promoting earlier heading. Vs treatment significantly inhibited young spikelet development, resulting in a significant delay in heading (Fig. 4C). For yield-related traits, compared to V treatment, Vs treatment significantly improved tiller number per plant, plant height, spike length, and fertile spikelet number, with an increase of 15.2% in grain number per spike (Fig. 4D and J). The spike width and TGW of the Vs group were slightly lower than those of the V group (Fig. 4K). The evolutionary trends of traits in most varieties were consistent with those of Ji 325. Except for Chinese Spring, Vs treatment promoted tillering and spike formation in most varieties by extending the vegetative growth period and inducing sufficient development of young spikes under low-temperature conditions (Table S9).

### 2.4 Low temperature affects yield-related traits by regulating plant hormone signal transduction pathways

Transcriptomic analysis revealed extensive reprogramming of gene expression in young spikes, especially in response to low-temperature treatment after the reproductive transition. We profiled the transcriptomes of Ji 325 young spikes during floret differentiation across V0, V, and Vs treatments. This identified 969 differentially expressed genes (DEGs) between V and V0, including 488 upregulated and 481 downregulated genes (Fig. S5A); 4103 DEGs between Vs and V0, with 3583 upregulated and 520 downregulated genes (Fig. S5B); and 4912 DEGs between Vs and V, with 3867 upregulated and 1045 downregulated genes (Fig. S5C). These results indicate that low-temperature treatment after the reproductive transition led to more significant changes in gene expression patterns in young spikes. To confirm the reliability of the transcriptomic data, we subjected eight DEGs to RT-qPCR validation: *VRN1* and *SVP3-B* (flowering-related); *MKKK62-B*, *ERF77*, *PP2C06*, *PP2C30-D*, and *SAPK9-A* (ABA signaling and stress-responsive); and *COR47-A* (cold-responsive). The expression patterns of all genes examined were highly consistent with the RNA-seq data across the three treatments (V, V0, and Vs) (Fig. S6), confirming the accuracy of the transcriptomic data.

**Fig. 5:**
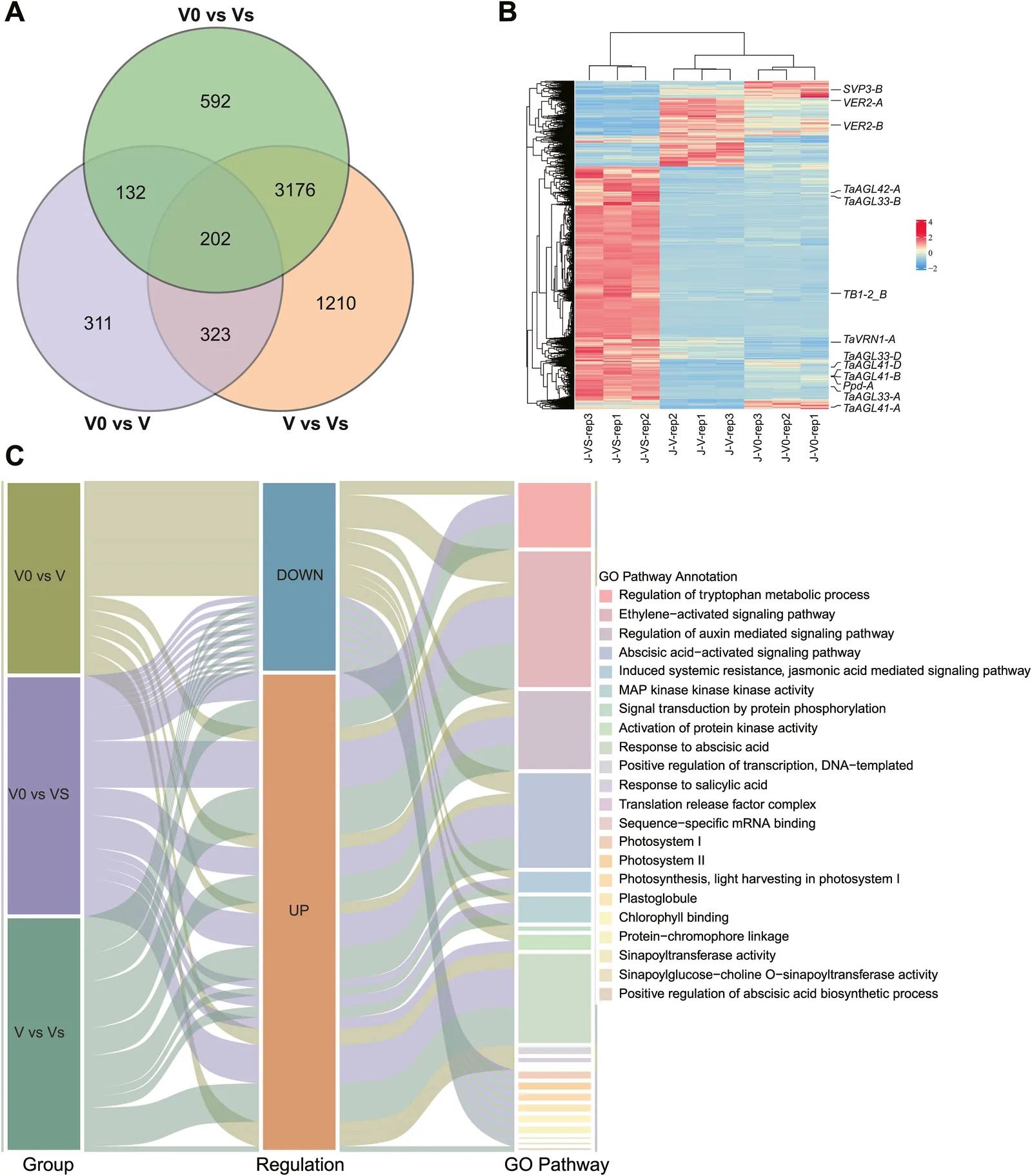
Post-transition low temperature triggers extensive hormone-centered transcriptional reprogramming in developing spikes. **A** Venn diagram showing the number of shared and unique DEGs between the three pairwise treatment comparisons (V0 vs. V, Vs vs. V0, and Vs vs. V). **B** Heatmap showing the expression patterns of genes; the dendrograms to the left of and above the heatmap show hierarchical clustering of the expressed genes and treatments, respectively. **C** Gene ontology (GO) enrichment analysis of regulated pathways under three different treatments; GO terms (biological process category) are listed on the right. V0: Without vernalization. V: Vernalization during the vegetative stage. Vs: Low-temperature treatment after the initiation of the reproductive transition.

Since some DEGs appeared in multiple comparisons, we further analyzed these genes. There were 968 DEGs between V and V0, 311 of which were differentially expressed depending on whether vernalization occurred during vegetative growth. In addition, 323 of these DEGs were differentially expressed under low-temperature treatment, including *TaAGL33* and *TaAGL41* (Fig. 5A and B). Moreover, 132 genes were differentially expressed depending on whether low-temperature treatment had been experienced, including *TaVRN1*, the key gene for vernalization (Fig. 5A and B). *AGL*, which is involved in the morphogenesis of inflorescences and floral organs, appeared in multiple comparisons (Fig. 5B). Compared to the V group, *TaAGL33*, *TaAGL41, TaAGL42*, *Ppd-B*, *TB1-2*, and *Ppd-A* were upregulated in the Vs group, whereas *VER2* was downregulated (Fig. 5B).

GO enrichment analysis revealed that, compared to the V0 group, low-temperature treatment at different stages induced significant changes in numerous biological processes (Fig. 5C and Table S10). Among the genes upregulated in Vs relative to V, multiple significantly enriched GO terms were identified, including genes associated with gibberellin degradation and ABA signaling pathways. A limited number of genes were differentially expressed between V0 and V, and these DEGs were primarily enriched in metabolic pathways, enzyme activities, and hormone signal transduction pathways. Compared to V0, Vs showed enrichment in multiple genes related to ABA biosynthesis and response pathways, as well as JA metabolism pathways (Fig. 5C). Further analysis of the metabolic pathways involving the DEGs revealed that, among the DEGs in Vs compared to V, besides numerous pathways associated with secondary metabolite synthesis, upregulated genes were also enriched in pathways involved in MAPK signaling and hormone signal transduction. Conversely, the downregulated genes were predominantly enriched in metabolism-related pathways. Perhaps vernalization during the seedling stage suppresses certain metabolic reactions while positively regulating specific plant hormone signal transduction pathways. Compared to V0, the upregulated genes in Vs were enriched in pathways related to secondary metabolite biosynthesis and pathways such as hormone signal transduction and biosynthesis. Low-temperature treatment at different stages might affect young spike development and yield-related traits by regulating the expression of genes related to hormone signal transduction pathways and flower development.

### 2.5 Temperature fine-tunes spikelet development by regulating the ABA signaling pathway

To investigate the effects of temperature treatment during young spike development on phytohormone levels and the underlying regulatory mechanisms, we measured the contents of the key phytohormones gibberellin (GA), abscisic acid (ABA), auxin, cytokinin (isopentenyladenine, iP), jasmonic acid (JA), and salicylic acid (SA) in young spikes of Ji 325 during the floret differentiation stage (Fig. S7 and Table S11). This analysis detected significant differences in phytohormone levels across the three treatment groups (Fig. S7A). ABA and SA levels exhibited dramatic variation, with ABA contents differing by nearly 20-fold (Fig. S7B and S7C). Overall, GA and auxin levels exhibited no significant differences (Fig. S7D and S7E), JA levels exhibited moderate differences (Fig. S7F), and the levels of cytokinin precursor substances increased markedly after vernalization treatment (Fig. S7G-L). These results suggest that low-temperature treatment at different stages regulates the expression of genes involved in the synthesis and signaling of key plant hormones such as ABA, SA and JA, thereby altering hormone levels and further influencing wheat spikelet development and yield-related traits, providing crucial insights into the molecular mechanisms of low-temperature regulated yield in wheat.

**Fig. 6:**
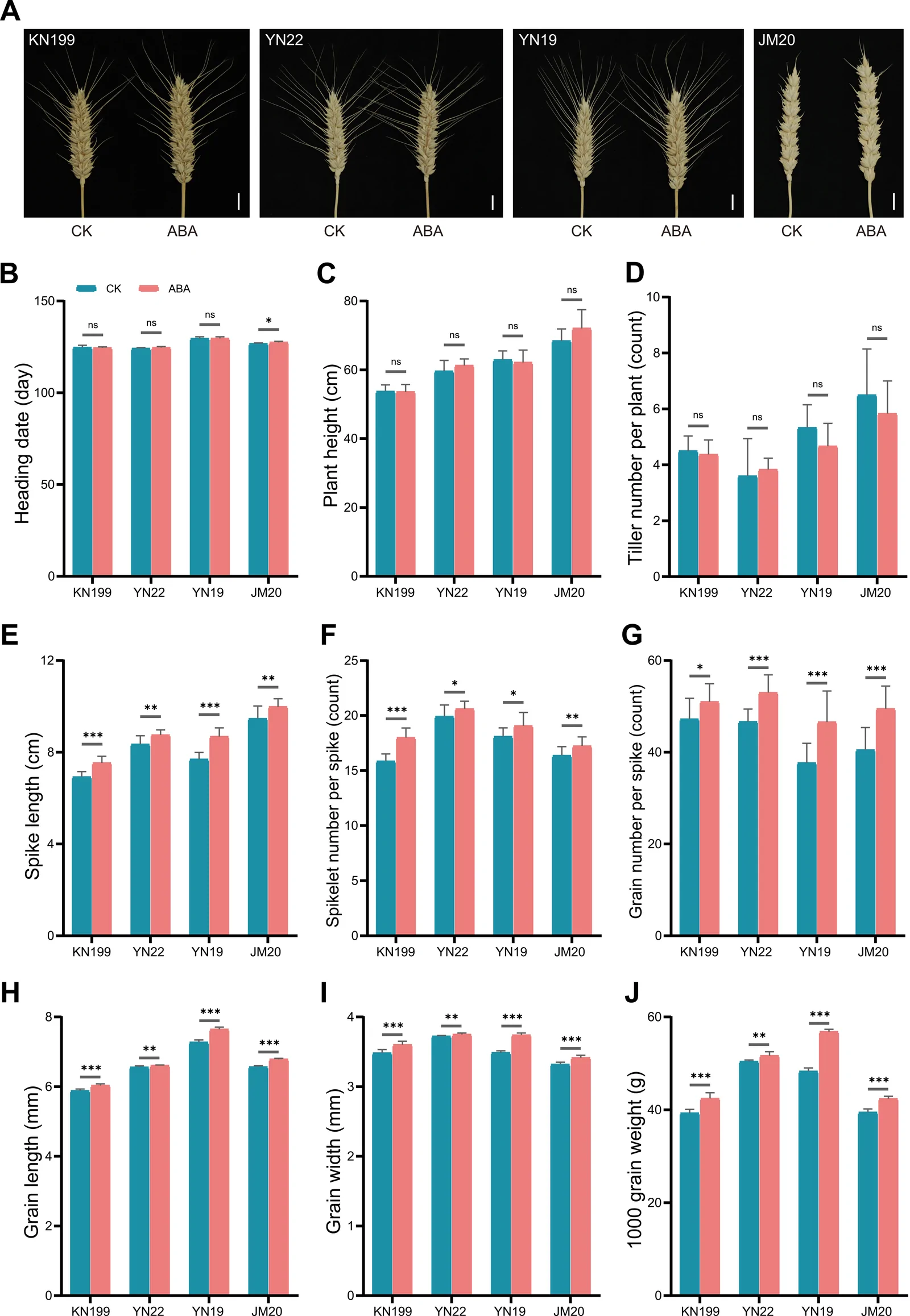
ABA treatment enhances spike development and yield-related traits in wheat. **A** Spike phenotypes of four wheat varieties (KN199, YN22, YN19, and JM20) after ABA treatment. CK: Control group; ABA: abscisic acid treatment group. Scale bar = 1 cm. **B-J** Heading date (**B**), plant height (**C**), tiller number per plant (**D**), spike length (**E**), spikelet number per spike (**F**), grain number per spike (**G**), grain length (**H**), grain width (**I**), and thousand grain weight (**J**) of four wheat varieties after ABA treatment. The data represent the mean ± SD; significant differences were analyzed by a two-tailed Student's *t*-test, ∗*P* < 0.05, ∗∗*P* < 0.01, ∗∗∗*P* < 0.001, ns: not significant.

To evaluate the effects of exogenous ABA on yield-related traits under low-temperature conditions, we transferred four representative wheat varieties (KN199, YN22, YN19, and JM20) to a growth chamber at 16 °C after the transition to reproductive growth and treated them with 10 μM ABA or diluted alcohol (CK). ABA treatment visibly promoted spike development compared with the control (Fig. 6A and Table S12). Although the heading date was largely unchanged, a slight but significant advancement in heading date was observed in JM20 (Fig. 6B). Quantitative analysis revealed that ABA treatment had no significant effect on plant height or tiller number per plant across all four varieties (Fig. 6C and D). Strikingly, ABA treatment significantly enhanced multiple yield-related traits. Spike length markedly increased in response to ABA in all four varieties (Fig. 6E). Similarly, spikelet number per spike significantly increased in response to ABA treatment in all genotypes (Fig. 6F). Grain number per spike showed a consistent, highly significant increase under ABA treatment across all four varieties (Fig. 6G). Moreover, ABA treatment significantly improved grain morphology and grain weight. Grain length and grain width increased in all varieties in response to ABA treatment (Fig. 6H and I). Consequently, TGW was significantly higher in the ABA-treated group than in the control for all genotypes (Fig. 6J).

Collectively, these results indicate that ABA treatment at 16 °C affects reproductive development, leading to increased spike size, grain number per spike, and TGW, thereby enhancing yield potential under low-temperature conditions.

## 3 Discussion

### 3.1 Low temperature at the reproductive stage boosts wheat yield

Wheat spike formation is a key factor determining final yield, a process highly dependent on environmental signals, particularly temperature [2]. Vernalization during the vegetative stage acts as an epigenetic switch, primarily mediated by *vernalization* (*VRN*) genes, to relieve the repression of flowering and permit the transition to reproductive growth [29,30]. Here, we demonstrated that low temperature after the reproductive transition functions as a morphogenetic modulator rather than as a trigger of phase change.

In this study, we combined sowing experiments in the field with precise temperature-controlled experiments in the greenhouse to systematically investigate the effects of low-temperature signals at different stages of growth on spike traits and final yield in wheat. Compared to traditional vernalization treatment during the vegetative growth phase [31], low-temperature treatment after the reproductive transition and during the early stage of panicle differentiation can more effectively optimize grain structure and increase grain number per spike, thereby enhancing yield potential (Fig. 1, Fig. 2). Low temperature can slow the rate of cell division and differentiation in developing young spikelets, allowing for longer periods of material accumulation and morphological establishment for the development of spikelets and florets [32]. This might be the physiological basis for the improvement of yield-related traits in wheat in response to low temperatures. Notably, this low-temperature response was dependent on genotype: the spring variety ‘Fielder’ experienced reduced yield under early sowing and low-temperature conditions due to its weaker cold tolerance (Fig. 1), whereas the semi-winter variety ‘Yannong 1212’ demonstrated good tolerance to late sowing (Fig. 1), which may be related to its lower sensitivity to temperature fluctuations during spike development. This indicates that genotype-dependent low-temperature perception and responses are the main reason for phenotypic changes under these conditions.

### 3.2 ABA-mediated crosstalk between low-temperature signals and spike development

Transcriptome analysis revealed that low temperatures during the vegetative stage and the panicle differentiation stage induce distinct gene expression patterns. The latter triggered more extensive transcriptional reprogramming, with highly significant enrichment of plant hormone signaling pathways, particularly those involved in ABA biosynthesis and signal transduction (Fig. 3, Fig. 5). This stage-specific responsiveness aligns with the observation that reproductive organs exhibit greater transcriptional plasticity in response to temperature cues compared to vegetative tissues [32]. During spikelet differentiation at low temperature, the ABA content in young spikelets increased by an order of magnitude (Fig. S6). Such rapid ABA accumulation in developing spikes at low temperature has also been documented in another study in wheat and in barley (*Hordeum vulgare*), where ABA functions as a developmental regulator rather than solely as a stress signal [33,34].

In vegetative tissues, cold-induced ABA accumulation typically triggers a survival strategy characterized by severe growth arrest, stomatal closure, and dormancy [35]. During vernalization, ABA biosynthetic genes such as *TaNCED* are significantly upregulated in leaves but exhibit minimal expression in the meristem itself, indicating that ABA is not a direct inducer of the floral transition [36]. In the current study, exposure to cold temperatures during the post-reproductive stage appeared to trigger extensive transcriptional reprogramming centered on ABA biosynthesis and signaling within spike tissues, with *PYL* family genes and the core SnRK2/PP2C signaling modules showing substantial upregulation (Fig. 3). This physiological function is fundamentally different from vernalization, which merely confers flowering competence, whereas ABA-mediated regulation during the reproductive stage directly shapes yield-related traits.

Temperature-gradient experiments further revealed that ABA pathway genes in young spikes at the reproductive stage exhibited peak expression at 16 °C, suggesting that ABA functions as a homeostatic rheostat that fine-tunes the developmental balance rather than merely serving as a stress alarm signal at this stage. Therefore, although ABA during vernalization only modulates cold memory at the vegetative phase, in the post-reproductive cold response pathway, ABA acts independently on spike organs to directly regulate young spike development and yield potential.

### 3.3 Multiple plant hormones synergistically regulate wheat spike development at low temperature

The temperature response of wheat is regulated by a coordinated network centered around ABA. Indeed, the expression of genes related to JA, SA, and auxin pathways was significantly induced under low-temperature conditions (Fig. S7). In cereals, endogenous plant hormonal signaling coordinately regulates floral meristem characteristics, activity, and determinism, particularly the fertility of florets [37]. Dynamic gene regulatory networks involving JA biosynthesis genes during the differentiation phase have been shown to improve spike fertility by regulating the fate of floret primordia [38]. Furthermore, the TaSPL13-2B-regulated JA signaling module (TaJAZ1-TaMYC2) contributes to floret fertility by inducing the expression of floral organ identity genes and upregulating genes involved in JA biosynthesis [39]. In the current study, genes involved in JA biosynthesis and signaling were upregulated during delayed vernalization treatment, and their expression levels were positively correlated with the number of fertile spikelets (Fig. 3, Fig. 5), suggesting that JA may participate in the determination of floret fate. In wheat, auxin homeostasis influences ABA biosynthesis and responses to abiotic stress, with crosstalk between IAA and ABA at the biosynthetic and developmental levels differentially affecting stress tolerance, depending on plant genotype [40]. Moreover, the activation of genes involved in auxin transport and responses may contribute to the expansion of vascular bundles and the efficient transport of assimilates to the inflorescence [41]. The differing responses to low temperatures among wheat ecotypes (such as winter and spring wheat) likely stem from variations in their phytohormone networks, particularly differences in the sensitivity or regulatory capacity of their ABA signaling systems. Further research is needed to explore the interactions between ABA and other plant hormones, as well as their specific regulatory pathways, during wheat growth and development.

Wheat plants that enter the reproductive stage too early may encounter cold winters, potentially causing developmental damage such as pollen abortion, whereas wheat entering this phase too late may miss the optimal window for low temperature–induced regulation. Enhancing the response efficiency of wheat to low temperatures through genetic modification might be a more effective approach than simply adjusting the sowing date. The key ABA signaling genes identified in this study could serve as targets for molecular marker-assisted selection, potentially helping breeders develop new wheat varieties that respond rapidly to low temperatures under late-sowing conditions while avoiding excessive cold sensitivity under early sowing. Our findings provide important theoretical and practical support for optimizing wheat sowing dates and breeding climate-resilient varieties, which could help ensure food security in light of global climate change.

## 4 Materials and methods

### 4.1 Plant material and growth conditions

On October 17 and November 4, 2021, representative wheat varieties were sown in the experimental field of Huazhong Agricultural University using a staggered planting design. The selected materials included the spring-type varieties Chinese Spring, Fielder, and Zhengmai 9023; the semi-winter-type varieties Ji 325, Ji 322, Ji 120, Yannong 1212, and Xinong 979; and the winter-type varieties Yannong 19, Jing 411, and Zhongmai 175. Each variety was sown in three rows with a spacing of 20 cm between rows, and a row length of 3 m. Seeds were sown using a drilling method in furrows uniformly opened to a depth of approximately 18 cm, with a plant spacing of about 10 cm and 1 or 2 seeds per hole. After sowing, a thin layer of soil was used to cover the seeds.

From January to May 2024, a natural population of 121 wheat accessions representing spring-type, semi-winter-type, and winter-type varieties were cultivated in the Rapid Breeding Platform at Huazhong Agricultural University. These accessions were globally sourced, have whole-genome sequences, and including landraces and improved cultivars. This diverse selection of materials allowed us to comprehensively investigate the responses of different types of wheat to temperature fluctuations during critical stages of spike development.

For ABA treatment, plants were transferred to a growth chamber at 16 °C under a 16-h light/8-h dark photoperiod after the transition to reproductive growth. The treatment group was sprayed with 10 μM abscisic acid solution, and the control group received an equal volume of diluted alcohol. Spraying was performed once every two days as previously described [42] until the plants reached maturity. The agronomic traits of the plants were evaluated at maturity.

### 4.2 Analysis of agronomic traits

To systematically evaluate the effects of temperature on yield-related agronomic traits in wheat, the following phenotypic parameters were measured for each accession under different sowing dates and temperature treatments: phenological traits, including heading date; plant morphological traits, including plant height and tiller number per plant; spike-related traits, including spike length, spikelet number per spike, and grain number per spike; and yield traits, including TGW. These traits were systematically analyzed to elucidate the adaptive responses of wheat at different developmental stages to temperature fluctuations and the underlying regulatory mechanisms governing final yield formation.

### 4.3 Experimental design of vernalization and temperature treatments for wheat materials

To investigate the differences in environmental temperature during young spike development caused by varying sowing dates, the wheat cultivar Ji 325 was grown in a greenhouse under three vernalization treatments: a non-vernalized control group maintained under ambient temperature conditions, a group vernalized during the vegetative stage after germination, and a group subjected to low-temperature treatment following the onset of the reproductive transition. Specifically, the non-vernalized control group was continuously grown at 22 °C under a 12-h light/12-h dark photoperiod. For the post-germination vernalization group, seedlings were transferred to a 4 °C cold room three days after germination and vernalized for four weeks under an 8-h light/16-h dark photoperiod (simulating short-day conditions), after which they were transferred to a 22 °C greenhouse with a 12-h light/12-h dark photoperiod for further growth. For the post-reproductive-transition vernalization group, plants were grown at 22 °C until the single-ridge stage, followed by low-temperature treatment at 4 °C for four weeks under an 8-h light/16-h dark photoperiod and a return to 22 °C under a 12-h light/12-h dark photoperiod until maturity.

Furthermore, to elucidate the adaptive strategies of wheat varieties under diverse environmental conditions, a natural population comprising 121 accessions was subjected to three temperature treatments, 8, 16, and 22 °C, for 14 days at the initial stage of the transition from vegetative to reproductive growth, after which they were returned to normal growth conditions to investigate the responses of wheat to different temperatures.

### 4.4 RNA extraction transcriptome analysis and qPCR

Young spike tissues were collected from wheat plants during the young spike differentiation stage (floret differentiation period) following the different treatments. Total RNA was extracted from the samples using a TransZol Kit (TransGen, ET121-01, China) following the manufacturer's instructions. RNA concentration and purity were assessed using a NanoDrop 2000 spectrophotometer (Thermo Fisher Scientific, USA). All RNA samples used for library construction met the following quality control criteria: an OD260/280 ratio between 1.8 and 2.1, and an OD260/230 ratio no less than 1.8.

RNA samples that passed quality inspection were used for cDNA library construction. The target sequencing data volume for each library was 20 Gb of raw data. After sequencing, the raw reads were processed to remove adapter-containing reads and low-quality reads, yielding high-quality clean reads. The reference genome sequence and annotation files used in this study were downloaded from the International Wheat Genome Sequencing Consortium RefSeq v1.1. Following read alignment, gene expression levels were quantified as fragments per kilobase of transcript per million mapped reads (FPKM).

Two micrograms of total RNA was reverse transcribed using a Vazyme Reverse Transcription Kit (R412-01) according to the manufacturer's instructions. The resulting cDNA was diluted four-fold with nuclease-free water, and 0.5 μL of the diluted cDNA was used in a 10 μL qPCR reaction. *TaActin* was used as the internal reference gene, and relative expression levels were calculated using the 2^-△△Ct^ method. Gene-specific primers used for qPCR are listed in Table S13.

### 4.5 Phytohormone extraction from young spikes

The plants used for phytohormone profiling were grown in the greenhouse alongside the plants used for transcriptomic analysis. Upon reaching approximately the seven-leaf stage, the leaves were removed, and the developmental stage of the young spikes was determined under a microscope. Young spike samples at the floret differentiation stage were collected and snap-frozen in liquid nitrogen. For the low-temperature treatment group, plants were subjected to 4 °C treatment immediately after the reproductive transition. After four weeks of treatment, the plants were returned to normal temperature conditions until the young spikes reached the floret differentiation stage for sampling. All samples were stored in an ultra-low temperature freezer at −80 °C until further use. Phytohormone extraction and analysis were performed as described previously [43].

### 4.6 Statistical analysis

Agronomic trait data were statistically analyzed using GraphPad Prism software; the results are presented as mean ± standard deviation (Mean ± SD). Prior to analysis of significant differences, tests for normality and homogeneity of variance were performed. Comparisons between two groups were conducted using a two-tailed Student's *t*-test. For multiple group comparisons, one-way analysis of variance (ANOVA) was performed, followed by Tukey HSD post-hoc test for multiple comparisons. The threshold for statistical significance was set at *P* < 0.05 (∗*P* < 0.05, ∗∗*P* < 0.01, ∗∗∗*P* < 0.001). All figures were generated using GraphPad Prism.

To explore the association between agronomic traits and molecular-level changes, Pearson correlation analysis was performed to calculate the correlation coefficients among agronomic trait parameters, plant hormone contents, and the expression levels (FPKM) of key genes, with statistical significance set at *P* < 0.05.

### 4.7 Construction and visualization of temperature-responsive transcriptional regulatory networks

To investigate the transcriptional regulatory mechanisms underlying temperature responses, a motif-supported transcriptional regulatory network was constructed based on the gene expression patterns of temperature-sensitive and temperature-insensitive materials treated at 8, 16, and 22 °C. Genes exhibiting temperature-dependent expression patterns were classified into six expression clusters (C1–C6) based on their expression pattern across different temperature conditions. For each cluster, promoter sequences spanning 3.5 kb upstream and 1.5 kb downstream of the annotated transcription start sites were extracted. TF-binding motifs were scanned using FIMO with a significance threshold of *q* < 1 × 10^−5^ to identify potential TF–target gene regulatory relationships. To improve the reliability of the predicted regulatory network, only TF–target pairs in which the upstream TF and target gene exhibited the same or consistent expression patterns within the corresponding material were retained. Based on these criteria, temperature-responsive candidate regulatory networks were constructed separately for temperature-sensitive and temperature-insensitive materials. Network topology analysis was performed by calculating the outdegree of each TF, representing the number of predicted downstream target genes, to identify potential regulatory hubs. The regulatory networks were visualized using Cytoscape (v3.10.4), and node size was scaled according to the number of target genes (outdegree) to highlight highly connected transcriptional regulators.

## CRediT authorship contribution statement

**Ankui Liu:** Writing – original draft, Investigation, Formal analysis, Data curation. **Siqi Wu:** Writing – original draft, Methodology, Investigation, Formal analysis. **Sulaiman Saeed:** Formal analysis, Data curation. **Mengxin Wang:** Formal analysis, Data curation. **Xindi Wang:** Formal analysis, Data curation. **Haotian Tu:** Formal analysis, Data curation. **Wenhao Yan:** Supervision, Project administration, Conceptualization. **Chao He:** Funding acquisition, Formal analysis, Data curation, Conceptualization. **Yequn Wu:** Writing – review & editing, Supervision, Project administration.

## Declaration of competing interest

The authors declare that they have no known competing financial interests or personal relationships that could have appeared to influence the work reported in this paper.

## Data Availability

The datasets generated during this study have been deposited in the Genome Sequence Archive (GSA) under accession number CRA042071.

## References

[1] Rezaei E.E., Webber H., Asseng S., Boote K., Durand J.L., Ewert F. (2023). Climate change impacts on crop yields. Nat Rev Earth Environ.

[2] Boden S.A., Cavanagh C., Cullis B.R., Ramm K., Greenwood J., Jean Finnegan E. (2015). *Ppd-1* is a key regulator of inflorescence architecture and paired spikelet development in wheat. Nat Plants.

[3] Jacott C.N., Boden S.A. (2020). Feeling the heat: developmental and molecular responses of wheat and barley to high ambient temperatures. J Exp Bot.

[4] Ding Y., Shi Y., Yang S. (2024). Regulatory networks underlying plant responses and adaptation to cold stress. Annu Rev Genet.

[5] Deng W., Casao M.C., Wang P., Sato K., Hayes P.M., Finnegan E.J., Trevaskis B. (2015). Direct links between the vernalization response and other key traits of cereal crops. Nat Commun.

[6] Lin F., Li C., Xu B., Chen J., Chen A., Hassan M.A. (2023). Late spring cold reduces grain number at various spike positions by regulating spike growth and assimilate distribution in winter wheat. Crop J.

[7] Weng Y., Tang Z., Huang W., Wang R., Wang F., Cai H. (2025). Integrated physiological and transcriptomic analyses reveal the mechanism of reduction in wheat fertile floret number under spring low-temperature stress. Crop J.

[8] Kiss T., Horváth Á.D., Cseh A., Berki Z., Balla K., Karsai I. (2024). Molecular genetic regulation of the vegetative-generative transition in wheat from an environmental perspective. Ann Bot.

[9] Liu Y., Liu P., Gao L., Li Y., Ren X., Jia J. (2024). Epigenomic identification of vernalization cis-regulatory elements in winter wheat. Genome Biol.

[10] Spanic V., Duvnjak J., Budimir K.S., Drenjancevic L., Jukic G., Varnica I. (2025). Measuring the effects of different sowing dates of winter wheat. Int J Plant Prod.

[11] Liu J., Shen Y., Zeng C., Zhang J., Shi S., Xue H. (2025). Effects of sowing time on yield and quality of winter and spring wheat varieties. Sustainability.

[12] Wen P., Wei Q., Zheng L., Rui Z., Niu M., Gao C. (2023). Adaptability of wheat to future climate change: effects of sowing date and sowing rate on wheat yield in three wheat production regions in the North China Plain. Sci Total Environ.

[13] Fujii H., Chinnusamy V., Rodrigues A., Rubio S., Antoni R., Park S.Y. (2009). In vitro reconstitution of an abscisic acid signalling pathway. Nature.

[14] Wang J., Sun L., Zhang H., Jiao B., Wang H., Zhou S. (2023). Transcriptome analysis during vernalization in wheat (*Triticum aestivum* L.). BMC Genom Data.

[15] Verma V., Ravindran P., Kumar P.P. (2016). Plant hormone-mediated regulation of stress responses. BMC Plant Biol.

[16] Zarea M.J. (2025). The regulatory roles of phytohormones in the wheat grain-filling process. J Plant Growth Regul.

[17] Su P., Sui C., Wang S., Liu X., Zhang G., Sun H. (2023). Genome-wide evolutionary analysis of *AUX/IAA* gene family in wheat identifies a novel gene *TaIAA15-1A* regulating flowering time by interacting with ARF. Int J Biol Macromol.

[18] Kosová K., Prášil I.T., Vítámvás P., Dobrev P., Motyka V., Floková K. (2012). Complex phytohormone responses during the cold acclimation of two wheat cultivars differing in cold tolerance, winter Samanta and spring Sandra. J Plant Physiol.

[19] Zhang W., Wang J., Huang Z., Mi L., Xu K., Wu J. (2019). Effects of low temperature at booting stage on sucrose metabolism and endogenous hormone contents in winter wheat spikelet. Front Plant Sci.

[20] Ahres M., Pálmai T., Farkas Z., Gulyás Z., Soltész A., Borbély P. (2025). Investigating the impact of spring (*Vrn-A1*) and winter (*vrn-A1*) vernalization alleles on frost tolerance induced by light spectrum and low temperatures in different wheat backgrounds. Environ Exp Bot.

[21] Wang X., Guan P., Xin M., Wang Y., Chen X., Zhao A. (2021). Genome-wide association study identifies QTL for thousand grain weight in winter wheat under normal- and late-sown stressed environments. Theor Appl Genet.

[22] Kanapickas A., Vagusevičienė I., Sujetovienė G. (2024). The effects of different sowing dates on the autumn development and yield of winter wheat in central lithuania. Atmosphere.

[23] Javed J., Zhang Z., Li J., Gou Q., Wang M. (2026). Pan-genomic characterization of the *NCED* genes and their response to abiotic stresses in wheat species. Curr Plant Biol.

[24] Wang Y., Wang L., Wang Y., Xia L., Li S., Dai K. (2025). Genome-wide characterization of the *Pyrabactin Resistance 1-like* (*PYL*) gene family in wheat and functional identification of *TaPYL2D*. Plant Sci.

[25] Yu X., Han J., Wang E., Xiao J., Hu R., Yang G. (2019). Genome-wide identification and homoeologous expression analysis of *PP2C* genes in wheat (*Triticum aestivum* L.). Front Genet.

[26] Mishra S., Sharma P., Singh R., Tiwari R., Singh G.P. (2021). Genome-wide identification and expression analysis of sucrose nonfermenting-1-related protein kinase (*SnRK*) genes in *Triticum aestivum* in response to abiotic stress. Sci Rep.

[27] Yang F., Sun X., Wu G., He X., Liu W., Wang Y. (2024). Genome-wide identification and expression profiling of the ABF transcription factor family in wheat (*Triticum aestivum* L.). Int J Mol Sci.

[28] Ai G., He C., Bi S., Zhou Z., Liu A., Hu X. (2024). Dissecting the molecular basis of spike traits by integrating gene regulatory networks and genetic variation in wheat. Plant Commun.

[29] Oliver S.N., Finnegan E.J., Dennis E.S., Peacock W.J., Trevaskis B. (2009). Vernalization-induced flowering in cereals is associated with changes in histone methylation at the *VERNALIZATION1* gene. Proc Natl Acad Sci.

[30] Xu S., Chong K. (2018). Remembering winter through vernalisation. Nat Plants.

[31] Cockram J., Jones H., Leigh F.J., O'Sullivan D., Powell W., Laurie D.A. (2007). Control of flowering time in temperate cereals: genes, domestication, and sustainable productivity. J Exp Bot.

[32] Yu X., Jiang Y., Yao H., Ran L., Zang Y., Xiong F. (2022). Cytological and molecular characteristics of delayed spike development in wheat under low temperature in early spring. Crop J.

[33] Ali F., Qanmber G., Li F., Wang Z. (2022). Updated role of ABA in seed maturation, dormancy, and germination. J Adv Res.

[34] Seiler C., Harshavardhan V.T., Rajesh K., Reddy P.S., Strickert M., Rolletschek H. (2011). ABA biosynthesis and degradation contributing to ABA homeostasis during barley seed development under control and terminal drought-stress conditions. J Exp Bot.

[35] Hirayama T., Shinozaki K. (2007). Perception and transduction of abscisic acid signals: keys to the function of the versatile plant hormone ABA. Trends Plant Sci.

[36] Cui G., Cheng H. (2025). Comprehensive analysis of *TaNCED* gene family in wheat vernalization process. Biology.

[37] Marzec M., Alqudah A.M. (2018). Key hormonal components regulate agronomically important traits in barley. Int J Mol Sci.

[38] Liu Y., Zhang L., Zhu A., Shen L., Zhang J., Chen J. (2025). Multi-omics identifies key genetic and metabolic networks regulating spike organ development in wheat. Plant Cell.

[39] Li L., Shi F., Wang Y., Guan Y., Wu Y., Chen L. (2026). Wheat *TaSPL13-2B* improves floret fertility and enhances grain number per spikelet through jasmonic acid signalling pathway. Plant Biotechnol J.

[40] Liu L., Yahaya B.S., Li J., Wu F. (2024). Enigmatic role of auxin response factors in plant growth and stress tolerance. Front Plant Sci.

[41] Moret B., Marhava P., Aliaga Fandino A.C., Hardtke C.S., Ten Tusscher K.H.W. (2020). Local auxin competition explains fragmented differentiation patterns. Nat Commun.

[42] Li Y., Jin L., Li W., Wang K., Su H., Mao H. (2025). Modulation of an evolutionarily conserved epigenetic regulon controlling abscisic acid catabolism enhances drought tolerance in wheat. New Phytol.

[43] Chen J., Hu X., Shi T., Yin H., Sun D., Hao Y. (2020). Metabolite-based genome-wide association study enables dissection of the flavonoid decoration pathway of wheat kernels. Plant Biotechnol J.

