# Supplementary material for: Low temperature modulates spike development and yield via ABA pathway at post-reproductive transition stage in wheat
Source: aBIOTECH. 2026 Aug 25;7(4):100086. doi: 10.1016/j.abiote.2026.100086 (PMC13582002; doi:10.1016/j.abiote.2026.100086)
Supplement: Supplementary file 1 [file mmc1.docx]

**Fig. S1.** Early sowing increases spikelet development and fertility-related spike traits. **A** Microscopic observation of young panicles of lately-sown plants under stable daily maximum temperature of 10°C, bar = 0.5mm; **B-D** Influence of sowing date on spikelet number per spike (**B**) fertile spikelet number (**C**) sterile spikelet number (**D**) in different wheat varieties. The data represent the mean ± SD, significant differences were determined by a two-tailed Student’s *t*-test, **P* < 0.05, ***P* < 0.01, ****P* < 0.001, ns: no significance. Each data point was derived from three sets of biological replicates, each containing ten samples.

 **Fig. S2.** Wheat varieties with different vernalization requirements exhibit distinct yield responses to temperature. **A** Heading date phenotype of representative varieties under different temperatures treatments, bar = 10cm. **B** Plant height of semi-winter, semi-spring, and spring varieties under different temperature treatments; **C** Grain number per spike of semi-winter, semi-spring, and spring varieties under different temperature treatments. The data represents the mean ± SD, significant differences were determined by one-way ANOVA, **P* < 0.05, ***P* < 0.01, ****P* < 0.001, ns: not significant.

 **Fig. S3.** Transcriptomic analyses reveal distinct temperature-responsive regulatory programs in sensitive and insensitive wheat genotypes. **A** Principal Component Analysis (PCA) plot showing clear separation among biological replicates of the three temperature treatments, Distinct separation between groups indicates significant transcriptomic differences induced by different treatments. **B** Correlation analysis of transcriptome profiles among different groups. Pearson correlation coefficients were calculated based on gene expression levels to evaluate the repeatability and correlation of biological replicates among different groups. The color scale represents the correlation coefficient (r), ranging from blue (low correlation) to red (high correlation). S means sensitive to temperature, I means insensitive to temperature. **C** Expression clustering of temperature-responsive genes in the insensitive wheat variety across 8°C, 16°C and 22°C. Genes with TPM < 0.5 at any temperature were excluded prior to clustering. **D** Top hub transcription factors identified from the motif-supported candidate regulatory network.

 **Fig. S4**. RT-qPCR validation confirms temperature-responsive expression of ABA-associated genes. **A-B** Relative expression of five selected genes (*MKKK70-A*, *bZIP62-D*, *HAP2G-B*, *PYL5*, and *SAPK10*) in sensitive (**A**) and insensitive (**B**) materials at 8°C, 16°C, and 22°C. Data were normalized to *TaActin* and are shown as means ± SD (n = 3). Significance was assessed by Student's t-test (**P* < 0.05; ***P* < 0.01; ****P* < 0.001; ns, not significant).

 **Fig. S5.** Low temperature after reproductive transition induces substantially greater transcriptional changes than vegetative-stage vernalization. **A-C** Volcano plot for three different treatments: V0 vs V (**A**), V0 vs Vs (**B**), and V vs Vs (**C**) respectively. Blue dots represent the genes with significant downregulation (negative log2 fold change and p value below the threshold). Red dots show genes with significant upregulation (positive log2 fold change and p value below the threshold). Gray dots display the genes that are not expressed significantly. V0: Without vernalization. V: Vernalization during vegetative stage. Vs: Low temperature treatment after initiation of reproductive transition.

 **Fig. S6.** RT-qPCR validation supports the reliability of transcriptomic responses to developmental-stage temperature treatments. **A–H** Relative expression levels of eight differentially expressed genes (*VRN1*, *SVP3-B*, *MKKK62-B*, *COR47-A*, *ERF77*, *PP2C06*, *PP2C30-D*, and *SAPK9-A*) were validated by qRT-PCR in three treatments (V, V0, and Vs). The expression data were normalized to the internal reference gene *TaActin* and are presented as means ± SD (n = 3). Significant differences were determined by Student's t-test and indicated by asterisks (**P* < 0.05; ***P* < 0.01; ****P* < 0.001; ns, not significant).

 **Fig. S7.** Hormone Content in Wheat Young Spikelet under Different Treatments. **A** Correlation analysis of hormone contents in three different treatments. High correlation coefficients (close to 1) indicate excellent repeatability among biological replicates. **B-L** Differences in content of abscisc acid (**B**) salicylic acid (**C**) gibberellin (**D**) auxin (**E**) jasmonic acid (**F**) cytokinin and intermediate products (**G-L**) in three different treatments. The data represents the mean ± SD, and the significance is estimated by one way ANOVA, **P* < 0.05, ***P* < 0.01, ****P* < 0.001, ns, not significant. Each data was derived from three sets of biological replicates.
